# Peripheral Frequency of CD4+ CD28− Cells in Acute Ischemic Stroke: Relationship With Stroke Subtype and Severity Markers

**DOI:** 10.1097/MD.0000000000000813

**Published:** 2015-05-22

**Authors:** Antonino Tuttolomondo, Rosaria Pecoraro, Alessandra Casuccio, Domenico Di Raimondo, Carmelo Buttà, Giuseppe Clemente, Vittoriano della Corte, Giuliana Guggino, Valentina Arnao, Carlo Maida, Irene Simonetta, Rosario Maugeri, Rosario Squatrito, Antonio Pinto

**Affiliations:** From the U.O.C di Medicina Interna e Cardioangiologia (AT, RP, DDR, CB, GC, VDC, CM, IS, AP), Dipartimento Biomedico di Medicina Interna e Specialistica (Di.Bi.M.I.S) University of Paler mo, Department of Maternal and Infant Health (AC), University of Palermo; Pronto Soccorso Unit (RP, RS), FondazioneIstituto S. Raffaele/Giglio of Cefalù; U.O.C di Reumatologia, Dipartimento Biomedico di Medicina Interna e Specialistica (Di.Bi.M.I.S) (GG), University of Palermo; and Department of Experimental Medicine and Clinical Neurosciences (VA, RM), University of Palermo, Palermo, Italy.

## Abstract

CD4+ CD28− T cells also called CD28 null cells have been reported as increased in the clinical setting of acute coronary syndrome. Only 2 studies previously analyzed peripheral frequency of CD28 null cells in subjects with acute ischemic stroke but, to our knowledge, peripheral frequency of CD28 null cells in each TOAST subtype of ischemic stroke has never been evaluated. We hypothesized that CD4+ cells and, in particular, the CD28 null cell subset could show a different degree of peripheral percentage in subjects with acute ischemic stroke in relation to clinical subtype and severity of ischemic stroke.

The aim of our study was to analyze peripheral frequency of CD28 null cells in subjects with acute ischemic stroke in relation to TOAST diagnostic subtype, and to evaluate their relationship with scores of clinical severity of acute ischemic stroke, and their predictive role in the diagnosis of acute ischemic stroke and diagnostic subtype

We enrolled 98 consecutive subjects admitted to our recruitment wards with a diagnosis of ischemic stroke. As controls we enrolled 66 hospitalized patients without a diagnosis of acute ischemic stroke. Peripheral frequency of CD4+ and CD28 null cells has been evaluated with a FACS Calibur flow cytometer.

Subjects with acute ischemic stroke had a significantly higher peripheral frequency of CD4+ cells and CD28 null cells compared to control subjects without acute ischemic stroke. Subjects with cardioembolic stroke had a significantly higher peripheral frequency of CD4+ cells and CD28 null cells compared to subjects with other TOAST subtypes. We observed a significant relationship between CD28 null cells peripheral percentage and Scandinavian Stroke Scale and NIHSS scores. ROC curve analysis showed that CD28 null cell percentage may be useful to differentiate between stroke subtypes.

These findings seem suggest a possible role for a T-cell component also in acute ischemic stroke clinical setting showing a different peripheral frequency of CD28 null cells in relation of each TOAST subtype of stroke.

## INTRODUCTION

In the late 1980s, Hansen and Martin described a subset of T CD4+cells with defective CD28 antigen called CD4+ CD28− cells also called CD28 null.^1–3^ This subset of T cells shows several characteristics of pathogenic cells and they are less susceptible to regulation by Treg CD4+ CD25 null cells^4,5^ and produce high amounts of γ-interferon and tumor necrosis alfa (TNF-α) hence becoming proinflammatory cells. Some studies reported that these types of cells have increased in the clinical setting of acute coronary syndrome (ACS).^4–6^

Previous findings by Liuzzo et al^4,7,8^ in subjects with ACS could suggest a relationship between T-cell activation and ischemic neuronal damage since a possible epidemiologic, pathogenic, and clinical parallel between ACS and acute cerebrovascular events such as acute ischemic stroke, plausibly suggesting a possible role for T CD4+ CD28− cells also in acute brain ischemia pathogenesis.

Only 2 studies previously analyzed peripheral frequency of CD28 null cells in subjects with acute ischemic stroke. The former conducted by Nadareishvili et al^9^ prospectively evaluated subjects with acute ischemic stroke analyzing the relationship between CD28 null cells and recurrence of a cerebrovascular event showing that high peripheral levels of CD28 null cells were significantly associated with event recurrence risk at 1-year follow-up. In a second study by Nowik et al,^10^ authors reported that CD4+ CD28 null cells were involved in mechanisms that increase stroke risk.

Thus, we hypothesized that CD4+ cells and, in particular, the CD4+ CD28− subset could show a different degree of increased peripheral percentage in subjects with acute ischemic stroke in relation to clinical subtype and severity of ischemic stroke.

The aim of this study was as follows:

1. to analyze peripheral frequency of CD4+CD28− cells in subjects with acute ischemic stroke in relation to TOAST diagnostic subtype;
2. to evaluate relationship of peripheral frequency of CD4+CD28− cells with scores of clinical severity of acute ischemic stroke,
3. to evaluate the ability of peripheral frequency of CD4+CD28− cells to predict stroke and its subtypes classified according TOAST classification.

## MATERIALS AND METHODS

We enrolled consecutive subjects admitted to our recruitment wards (ward of Internal Medicine, AOUP “P.Giaccone” Palermo; ward of Vascular Medicine AOUP “P.Giaccone”, Pronto Soccorso Unit, Fondazione Istituto S. Raffaele/Giglio of Cefalù) with a diagnosis of acute ischemic stroke, in a recruitment period from June 2011 to December 2013. As controls we enrolled hospitalized patients without a diagnosis of acute ischemic stroke, admitted in the same period to our Internal Medicine Ward for any cause other than acute cardiovascular and cerebrovascular events. All enrolled patients underwent a general and neurological evaluation and an instrumental evaluation (ECG, ECG-holter 24 hours, epicranial vessel echography, brain CT or MRI, transthoracic echocardiography, and, in some cases, transesophageal).

Ischemic stroke has been defined as “a clinical syndrome of rapidly developing symptoms or signs of focal loss of cerebral function with symptoms lasting more than 24 hours and no apparent cause other than the vascular origin.”^11^ Patients and controls were excluded if they had 1 of the exclusion criteria: rheumatologic disorders, chronic inflammatory disease, acute systemic infections, recent venous thrombosis, recent acute myocardial infarction (AMI) (within 3 months), and recent cerebrovascular event (TIA or stroke within 6 months) (all these conditions may influence inflammatory cytokine and cell levels).

## CRITERIA FOR EVALUATION OF CARDIOVASCULAR RISK FACTORS FOR CASES AND CONTROLS

Type 2 diabetes mellitus was determined using a clinically based algorithm that considered age at onset, presenting weight and symptoms, family history, onset of insulin treatment, and history of ketoacidosis. Hypertension was defined according to the 2007 European Society of Hypertension and the European Society of Cardiology Guidelines (2007 ESH/ESC 2007) as follows: (i) systolic blood pressure (SBP) >140 mmHg, and/or diastolic blood pressure (DBP) >90 mmHg in patients not receiving antihypertensive medication; (ii) previously documented diagnosis of hypertension in patients with the concurrent use of diet or antihypertensive medication regardless of current SBP and DBP values. Hypercholesterolemia was defined as total serum cholesterol ≥200 mg/dL and hypertriglyceridemia as total serum triglyceride ≥150 mg/dL on the basis of the National Cholesterol Education Program–Adult Treatment Panel III reports that define this cutoff for optimal total serum cholesterol and triglyceride levels. All patients had blood pressure, serum glucose, creatinine, serum uric acid, serum cholesterol levels, serum triglyceride levels, and urinary albumin excretion values measured on admission to the hospital. Coronary artery disease was identified on the basis of a history of physician-diagnosed angina, myocardial infarction, or any previous revascularization procedure determined by a questionnaire. Cerebrovascular disease (TIA/ischemic stroke) was identified by patient history, specific neurologic examination performed by specialists, and hospital or radiological records (brain computed tomography or brain magnetic resonance) of definite TIA or stroke. The protocol study was approved by Ethics Committee of the Policlinico P Giaccone Hospital and of Fondazione Istituto S. Raffaele/Giglio of Cefalù and all the patients gave their written informed consent to participate in the study, as well as for sampling and banking of the biological material. Study protocol conforms to the ethical guidelines of the 1975 Declaration of Helsinki.

## BLOOD SAMPLE COLLECTION TIMING

Blood samples has been drawn after 48 hours after symptom onset owing to the fact that several reports from experimental models to humans^12,13^ can sustain a possible “peripheral blood translation” of a neuroinflammatory cascade, either in terms of inflammatory cytokines or in terms of “cellular trafficking,” so it is plausible to hypothesize a peripheral increase of some cell subset of the T-cell population within 48 hours after an acute ischemic cerebral event. Activated T-cells on the periphery of the immune compartment once recruitment by means of cytokines has been fulfilled, enter the cerebral level through blood brain barrier (BBB) disruption and thus it appears plausible to expect a higher frequency of some T-cell subsets on peripheral blood^14,15^ in parallel with their course of intracerebral inflow

### Cell Isolation, Staining, and Flow Cytometry

Peripheral blood has been drawn at 48 hours after symptom onset and after informed consent had been obtained from the patient or his/her authorized representative. Peripheral blood mononuclear cells (PBMCs) have been obtained by density gradient centrifugation using the lymphocyte separation medium (ICN Pharmaceutical, Costa Mesa, CA). This protocol yields an average PBMC composition of 60% T cells, 15% monocytes/macrophages, 10% B cells, and 15% natural killer cells.^16^ White cells were obtained from 2 mL of peripheral EDTA-anticoagulated venous blood. Cells were labeled with human monoclonal anti-CD4 antibodies conjugated with fluorescein isothiocyanate and anti-CD28 antibodies conjugated with phycoerythrine (Becton, Dickinson and Company 1 Becton Drive Franklin Lakes, New Jersey). Mouse IgG1 antibodies conjugated with fluorescein isothiocyanate (IgG1-FITC) and IgG2a conjugated with phycoerythrine IgG2a-PE) (Becton Dickinson) were used as the isotype controls. The samples were incubated for 30 minutes in the dark at ambient temperature, washed with 5% saline and centrifuged. The pellet was suspended in 1% formalin. Expression of the CD28 receptor on lymphocytes was studied with a FACSCalibur flow cytometer (FACSCalibur/Sysmex XT1800i dual platform) operating with CellQuest OS2 software. The population of CD4+ CD28− was expressed as a percentage of CD4+ cells (CD4+ CD28− and CD4+ CD28+).

## STROKE SUBTYPE EVALUATION

The type of acute ischemic stroke was classified according to the TOAST classification^17^: large artery atherosclerosis (LAAS); cardioembolic infarct (CEI); LACunar infarct (LAC); stroke of other determined etiology (ODE); and stroke of undetermined etiology (UDE).

## FUNCTIONAL EVALUATION

National Institute of Health Stroke Scale (NIHSS) and Scandinavian Stroke Scale (SSS) were used to evaluate acute neurological deficit grade at 48 hours after admission in all enrolled patients with acute ischemic stroke.^18,19^ Modified Rankin score (mRankin) was used to assess disability grade at discharge.^20^

### Statistical Analysis

Statistical analysis of quantitative and qualitative data, including descriptive statistics, was performed for all items. Continuous data are expressed as mean ± standard deviation, unless otherwise specified. Baseline differences between groups were assessed by the χ^2^ test or Fisher exact test as needed for categorical variables, and by the independent Student *t* test for continuous parameters. The univariate analysis of variance (ANOVA) was performed for parametric variables, and post hoc analysis with the Bonferroni test was used to determine whether there were pairwise differences. Linear regression analysis examined the correlation between various patient characteristics (independent variables), and CD4+ cells or CD4+ CD28− cells peripheral percentages (dependent variable) in simple and multiple regression models; at multivariate analysis we analyzed relationship between prognostic indexes (SSS, NIHSS, and rankin scores and death) and CD4+ and CD28 null cell peripheral percentage after adjustment for other variable resulted significant at univariate analysis. To assess the predictive rate of different cutoff values of CD4 or CD4CD28 peripheral percentages with regard of stroke and TOAST subtype, a receiver operating characteristic (ROC) curve with calculations of area under the curve and 95% CIs was constructed and sensitivity and specificity values were calculated. Data were analyzed by the Epi Info software (version 6.0, Centers for Disease Control and Prevention, Atlanta, GA) and IBM SPSS Software 21.0 version (SPSS, Inc., Chicago, IL). All *P* values were 2-sided and *P* values <0.05 were considered statistically significant.

## RESULTS

We enrolled 98 subjects with acute ischemic stroke and 66 control subjects. Demographic, laboratory, and clinical variables of subjects with acute ischemic stroke and control subjects are reported in Table 1.

**Table 1:**
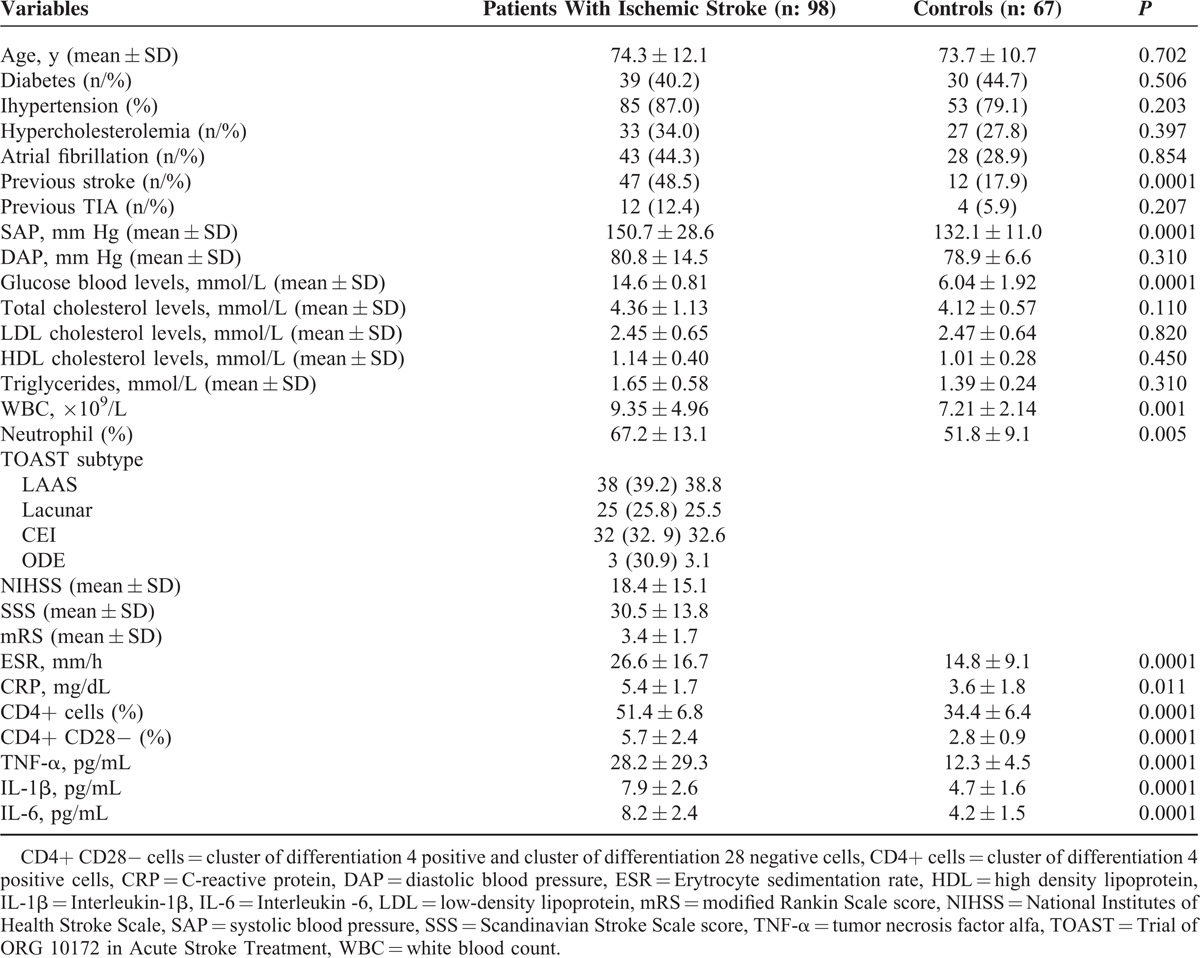
General, Clinical, and Laboratory Variables of Patients With Acute Ischemic Stroke (n: 98) and Controls (n: 67)

Subjects with acute ischemic stroke had a significantly higher peripheral frequency of CD4+ cells compared to controls without acute ischemic stroke (51.4 ± 6.8% vs 34.4 ± 6.4%; *P* = 0.0001); similarly, ischemic stroke subjects had a significantly higher peripheral frequency of CD4+CD28− cells compared to controls without acute ischemic stroke (5.7 ± 2.4% vs 2.8 ± 0.9%; *P* = 0.0001). Stroke subjects also showed higher cytokine plasma levels such as TNF-α **(**28.2 ± 29.3 vs 12.3 ± 4.5 pg/mL; *P* = 0.0001), IL-1β (7.9 ± 2.6 vs 4.7 ± 1.6 pg/mL; *P* = 0.0001) and IL-6 (8.2 ± 2.4 vs 4.2 ± 1.5 pg/mL; *P* = 0.0001) compared to controls (see Table 1).

Demographic, clinical and laboratory variables of subjects with acute ischemic stroke in relation to TOAST subtype are listed in Table 2.

**Table 2:**
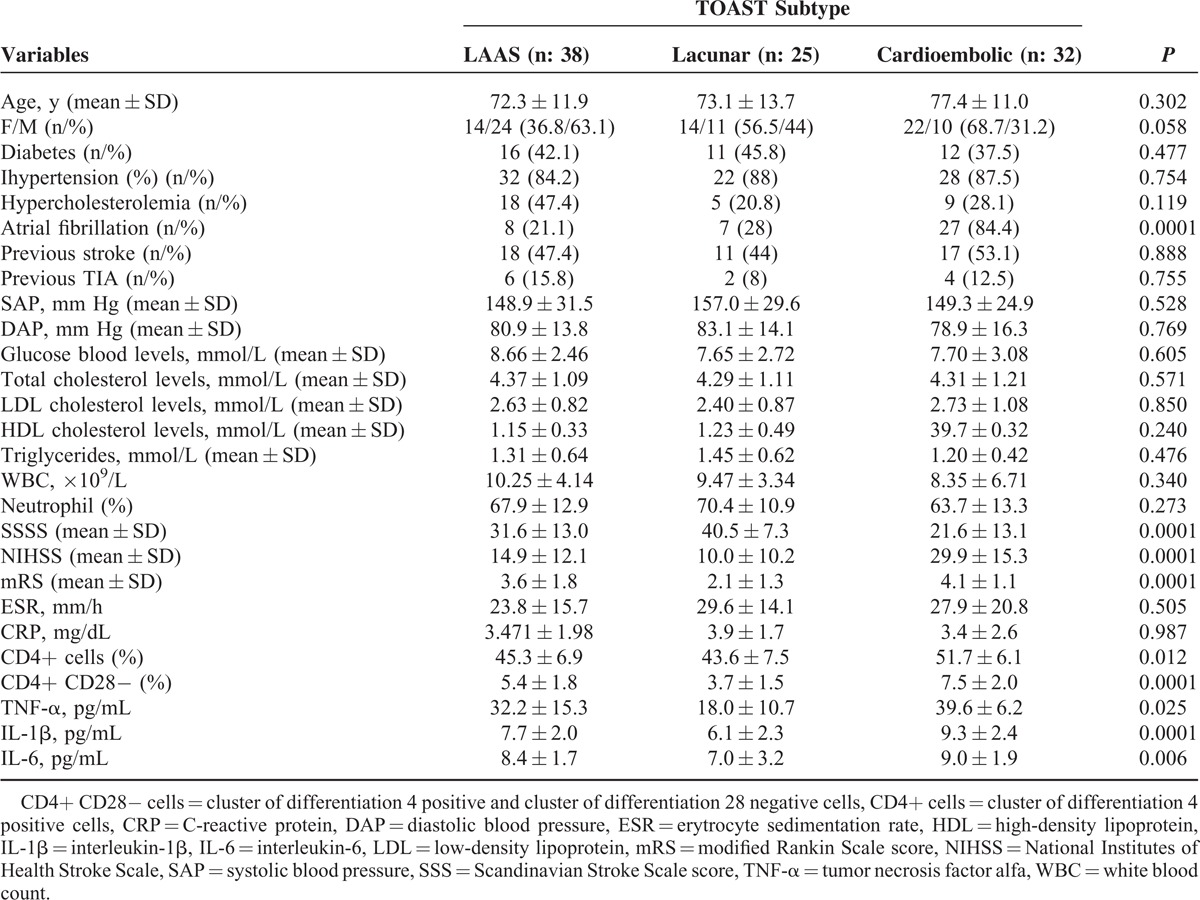
General, Clinical, and Laboratory Variables of Patients With Acute Ischemic Stroke in Relation of TOAST Subtype

Subjects with cardioembolic stroke showed a significantly higher peripheral frequency of CD4+ cells compared to subjects with LAAS and lacunar subtype (51.7 ± 6.1% vs 45.3 ± 6.9% vs 43.6 ± 7.5%; *P* = 0.012) and of CD28 null cells compared to subjects with LAAS and lacunar subtypes (7.5 ± 2.0% vs 5.4 ± 1.8% vs 3.7 ± 1.5%; *P* = 0.0001). Subjects with cardioembolic subtype had significantly higher blood levels of TNF-α (39.6 ± 6.2 vs 32.2 ± 15.3 vs 18.1 ± 10.7 pg/mL); IL-6 (9.03 ± 1.90 vs 8.44 ± 1.73 vs 7.04 pg/mL; *P* = 0.006) and IL-1β (7.7 ± 2.0 vs 6.1 ± 2.3 vs 9.3 ± 2.4 pg/mL) compared to subjects with LAAS and lacunar subtypes (see Table 2).

Regarding the relationship between immune-inflammatory variables and severity markers (see Table 3), we have observed no significant relationship between peripheral frequency of CD4+ cells and chosen stroke severity indicators, whereas we observed a significant relationship between peripheral frequency of CD4+CD28− cells and SSS (β = −0.049; *P* = 0.046) and NIHSS (β = −0.460; *P* = 0.042) scores.

**Table 3:**
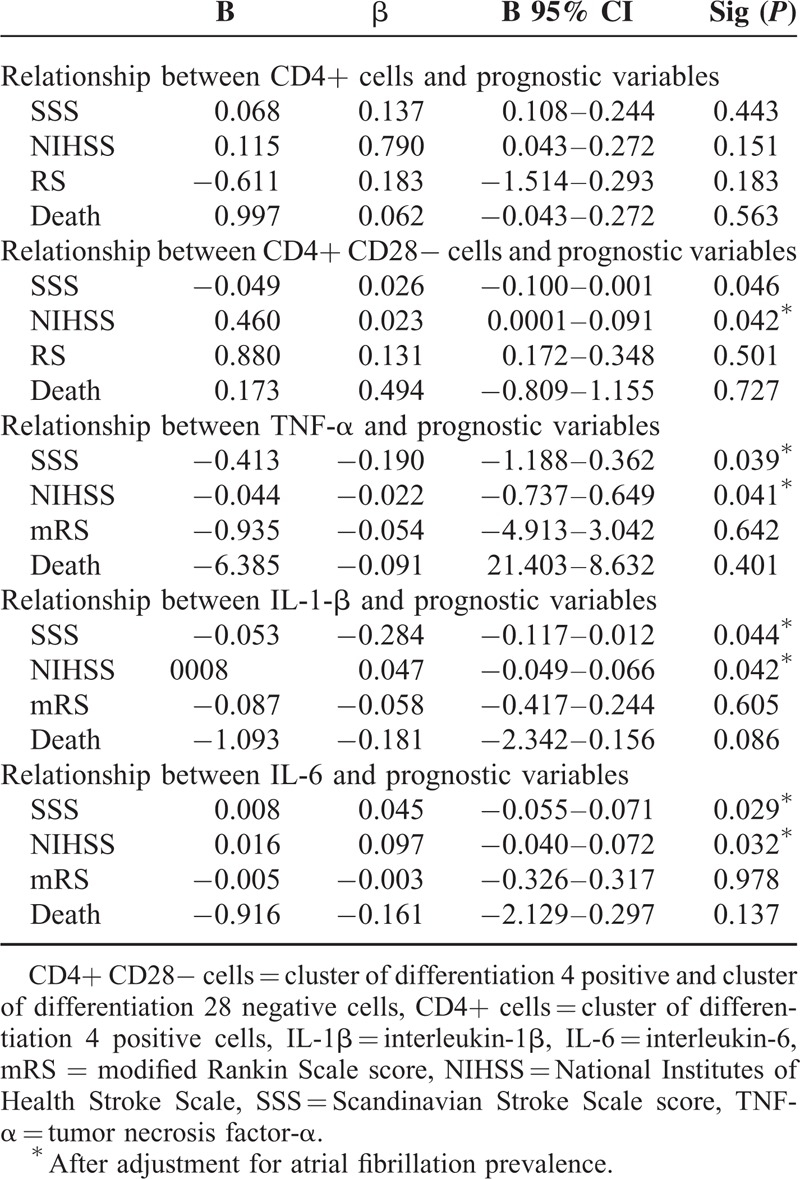
Multivariate Analysis of Relationship Between Immunoinflammatory and Prognostic Variables

At multivariate analysis, we reported a significant relationship between TNF-α (*P* = 0.039 and *P* = 0.041), IL-1β (*P =* 0.044 and *P* = 0.042), IL-6 plasma levels (*P* = 0.029 and *P* = 0.032), SSS and NIHSS scores, whereas CD28 null cell peripheral frequency was significantly associated with inflammatory cytokine blood levels at multivariate analysis, such as IL-6, IL-1β, and TNF-α (see Tables 4 and 5).

**Table 4:**
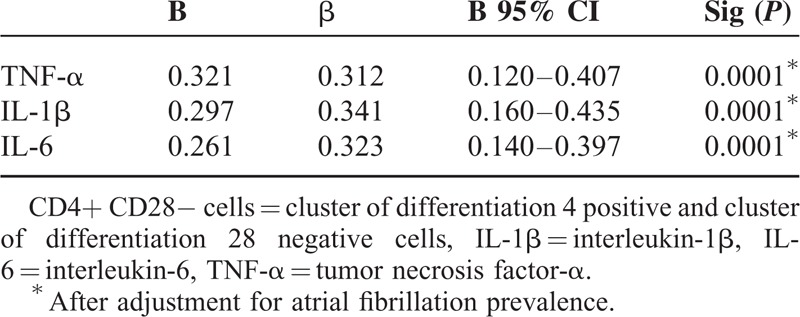
Multivariate Analysis of Relationship Between CD4+ CD28− Cells and Immunoinflammatory Variables

**Table 5:**
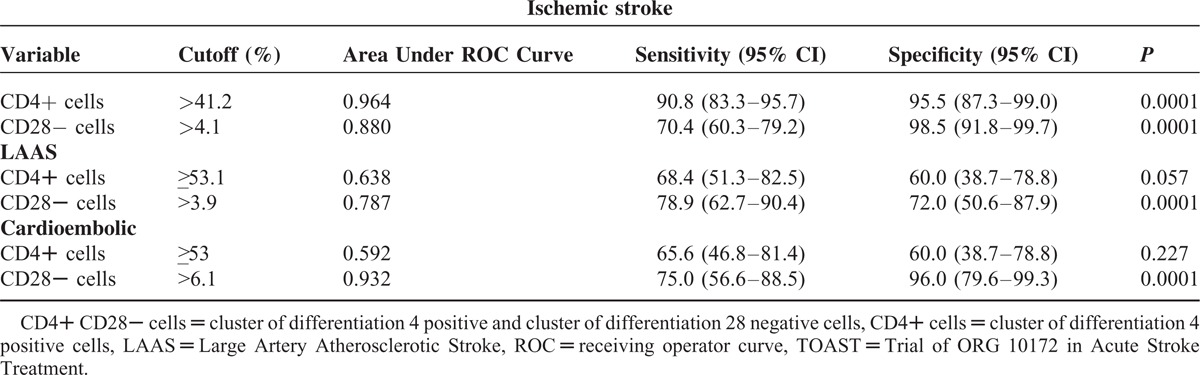
Area Under ROC Curve, Sensitivity, and Specificity of CD4+ and CD28− Cells Cutoff Values Diagnosis of Ischemic Stroke and TOAST Subtype

**FIGURE 1:**
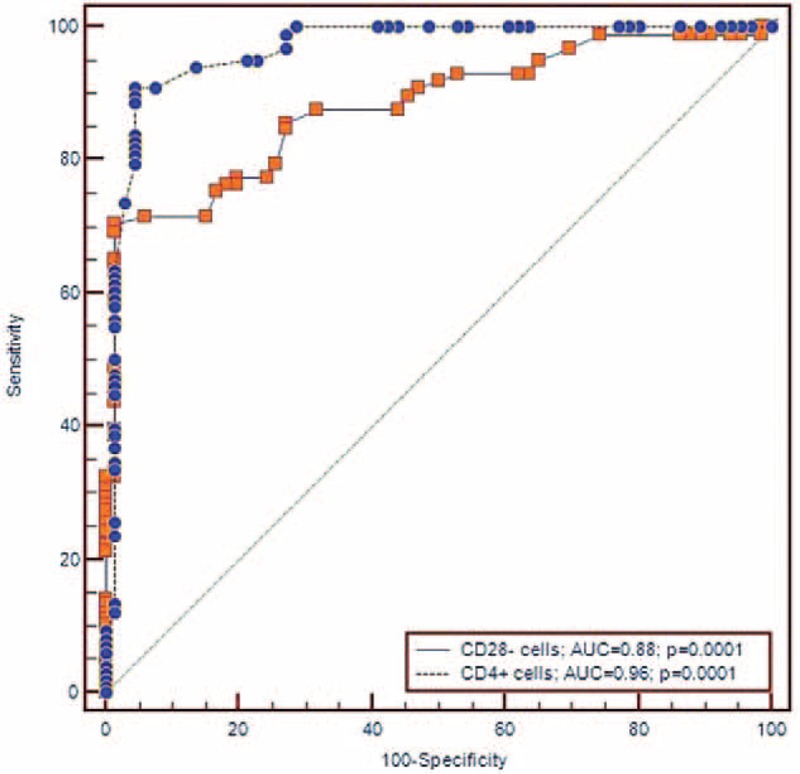
ROC curves of CD4+ and CD28− cells cutoff values toward diagnosis of ischemic stroke.

By means of ROC curve analysis we showed a good sensitivity and specificity of CD4+ peripheral frequency to predict ischemic stroke (AUC = 0.964, *P* = 0.0001; cutoff value > 41.2%, sensitivity = 90.9, specificity = 95.5; see Figure 1); in regard to the peripheral frequency of CD28− cells ROC curve analysis demonstrated good sensitivity and specificity to predict stroke (AUC = 0.880, *P* = 0.0001; sensitivity = 70.4, specificity = 98.5, cutoff value > 4.1%, (see Figure 1).

The sensitivity and specificity of CD4+ cells to predict TOAST subtype of ischemic stroke at ROC curve analysis did not show a significant association with LAAS (AUC = 0.638, *P* = 0.06; sensitivity = 68.4, specificity = 60.0 cutoff ≥53.1%), and cardioembolic subtypes (AUC = 0.592, *P* = 0.227; sensitivity = 65.6, specificity = 60.0, cutoff value ≥53).

**FIGURE 2:**
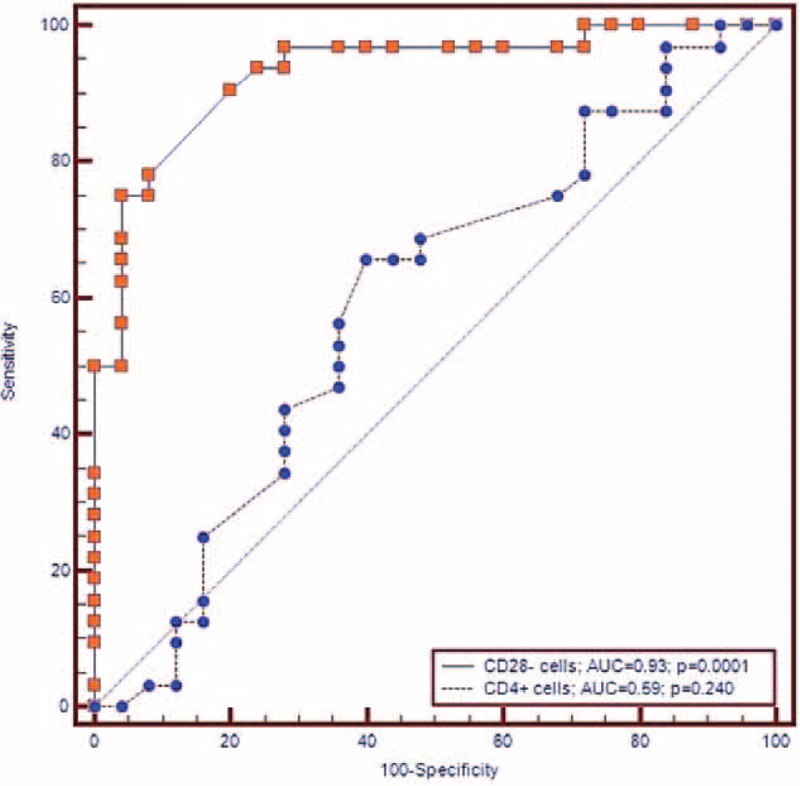
ROC curves of CD4+ and CD28− cells cutoff values toward diagnosis of cardioembolic subtype of ischemic stroke.

**FIGURE 3:**
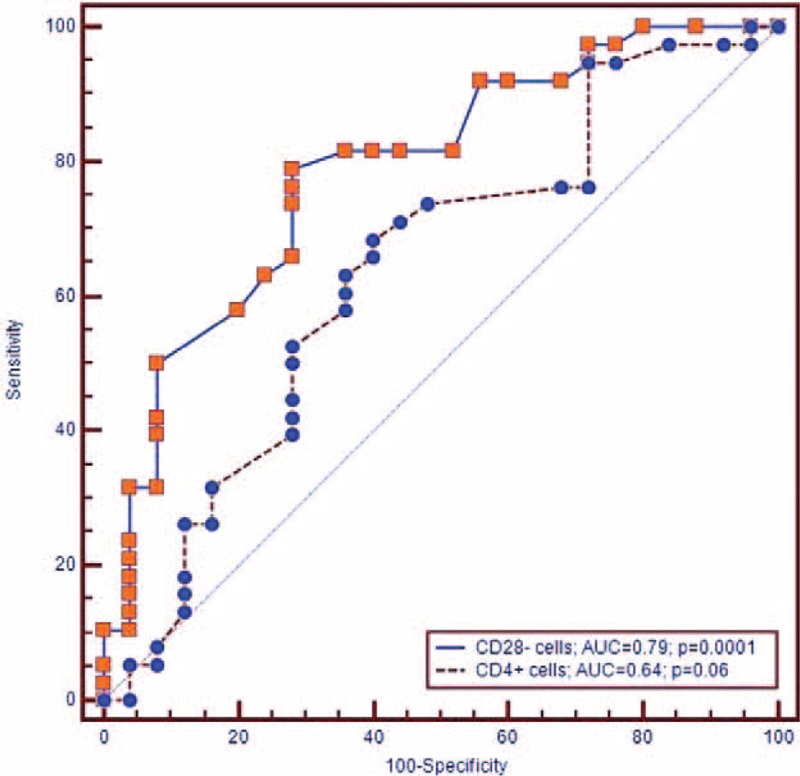
ROC curves of CD4+ and CD28− cells cutoff values toward diagnosis of LAAS subtype of ischemic stroke.

ROC curve analysis showed good sensitivity and specificity values of CD28 null cell peripheral frequency to predict cardioembolic (AUC = 0.932, *P* = 0.0001; sensitivity = 75.0, specificity = 96.0, cutoff value > 6.1%), and LAAS TOAST subtypes (AUC = 0.787, *P* = 0.0001; sensitivity = 78.9, specificity = 72.0, cutoff value > 3.9%) (see Figures 2 and 3).

## DISCUSSION

This study found a significantly higher peripheral frequency of CD4+ and CD28− cells in subjects with acute ischemic stroke compared to controls. Consistent with findings already reported by previous studies in the clinical setting of ACSs^4,7,8^ our results seem to suggest a possible role for a T-cell component also in ischemic stroke setting.

The acute phase of ischemic stroke is characterized by a high degree of immune-inflammatory activation in terms of increased plasma levels of cytokines, adhesion molecules, and selectins,^21,22^ and our group previously reported this degree of inflammatory activation as higher in subjects with cardioembolic subtype compared to the other TOAST subtypes of stroke.^12,13,14,15,23–26^

Several reports from experimental models to humans^16,27^ can sustain a possible “translation” of a neuroinflammatory cascade, either in terms of inflammatory cytokines or in terms of “cellular trafficking,” so it is plausible to hypothesize a peripheral increase of some cell subset of the T-cell population within 48 hours after an acute ischemic cerebral event. Activated T-cells on the periphery of the immune compartment once recruitment by means of cytokines has been fulfilled, enter the cerebral level through BBB disruption and thus it appears plausible to expect a higher frequency of some T-cell subsets on peripheral blood^16,27^ in parallel with their course of intracerebral inflow.

Among these T-cells, CD28 null cells that show several characteristics of pathogenic cells and they are less susceptible to regulation by Treg CD4+ CD25 null cells^4,5^ and produce high amounts of γ-interferon and TNF-α thus may have a direct pathogenetic role in neuronal ischemic damage.

Our finding concerning a significant relationship between T-cell subsets and TOAST subtype of stroke showing a higher peripheral frequency of CD4+ and CD28− cells in subjects with cardioembolic subtype compared to lacunar subtype appear original owing to the fact that to our knowledge, no study has yet addressed this issue.

At multivariate analysis our findings also showed a significant relationship between peripheral frequency of CD28 null cells and some severity markers such as SSS and NIHSS scores. Two previous studies, the former by Nadareishvili et al^9^ did not evaluate the relationship between neurological deficit grade and CD4CD28 null cell count, whereas Nowik et al^10^ reported that the severity of neurological deficits assessed on admission did not correlate with percentage of CD4+CD28− lymphocytes. It explains how our observed relationship between CD4+CD28 null cell peripheral percentage and NIHSS and SSS scores appear a novel finding.

Ischemic stroke induces a profound local inflammatory response. Within hours, various types of immune cells transmigrate over the activated endothelium to invade the damaged brain in a timed fashion. Although previous studies mostly focused on neutrophils and monocytes,^16,28,29^ the role of lymphocytes, especially T cells in ischemic stroke, has long been neglected, although T cells are localized in close vicinity to blood vessels in the infarct boundary as early as 24 hours after experimental focal cerebral ischemia in rodents.^30,31^ T cells have been identified in the brain as early as 24 hours after ischemia.^32,33^ Involvement of adaptive immunity comes from studies on the role of lymphocytes in models of focal cerebral ischemia reporting how ischemia leads to infiltration of the major lymphocytes subtypes into the ischemic brain.^33^

Lymphocytes invade the ischemic brain and contribute to tissue damage, but the rapidity of their deleterious effect is not consistent with an adaptive immune response targeted to the brain. Nevertheless, this lymphocyte role in brain ischemia pathogenesis could offer biological plausibility to our findings owing to the proinflammatory properties of CD28 null cells^4,5^

We previously reported that immune-inflammatory activation of the acute phase of ischemic stroke is associated with stroke volume and severity degree in terms of acute neurological deficit grade evaluated by NIHSS,^23^ thus the role of CD4+ CD28− subset could represent a natural extension of cytokine, selectins, and adhesion molecule activation. The severity of neurological symptoms, assumed to reflect the size of the ischemic lesion, correlated with the percentage of CD4+ CD28− lymphocytes. According to the hypothesis that stroke is followed by an increase in the number of these cells as a response to antigens released from injured brain tissue, a higher percentage of peripheral CD4CD28 null cells could be related to a more profound brain injury.

The possible neuroinflammatory equivalence between CD4+ CD28− cell components and cytokine activation just reported by our group^14,23^ and other groups^21,22^ is further confirmed by our finding concerning the relationship between frequency of peripheral CD28 null cells and some severity markers such as the SSS score and the NIHSS, used as indicators of the degree and type of neurological deficit of the acute phase. The significant association between levels of CD28− T cells and serum levels of inflammatory cytokines assessed in our current study (IL-6, TNF-α, IL-1β) may explain this relationship.

Finally, at ROC curve analysis our findings showed that CD28 null cell peripheral percentage may be useful to differentiate between stroke subtypes. The greater frequency of CD4+ CD28− in subjects with ischemic stroke compared to controls and the significant association with the degree and type of neurological deficit and with the cardioembolic subtype offers the possibility to analyze a possible application of the peripheral levels of the T-cell subset for differentiate stroke and its diagnostic subtype.

The greater association with the cardioembolic subtype emphasizes the role of a relationship between increased gravity and higher extent of cardioembolic stroke lesions^23^ and the activation of a subset of T cells such as the CD4+ and CD28− cells compared to the related component of the single plaque instability, than has been indicated by the few other studies that have addressed this issue.^4–6^

Furthermore, good sensibility and specificity for stroke diagnosis could offer prospects both in the diagnosis of a disease such as acute ischemic stroke that often enters the differential diagnosis with other acute illnesses, and for which a battery of diagnostic markers with sufficient sensitivity and specificity is not available, and for which the assessment of T-cell activation as well as an evaluation of the cytokine activation pathway could have a possible role in the integration and implementation of the diagnostic process.

This study has some limitations. First, we do not have any information about CD28 null peripheral percentage in baseline conditions prior of stroke occurrence. Second, CD 28 null peripheral percentage could better represent a proinflammatory background linked to stroke pathogenesis that a consequence of brain ischemic event. Third, our stroke patients showed a significantly higher prevalence of a previous stroke compared to controls thus, this higher previous cerebrovascular morbidity, could be linked to the higher degree of CD4+CD28− peripheral percentage in stroke patients. Fourth, some studies^34,35^ reported a finding of lymphopenia after acute ischemic stroke, but consistent with our findings other studies reported a higher frequency of some T-cell subsets on peripheral blood^14,15^ after an acute ischemic stroke underlyng the role of CD4CD28 null cells that are only a component of lymphocyte cell family. The profound damage to the CNS caused by ischemic lesions has been well documented.

Yet, relatively little is known about the contribution to and effects on the immune system during stroke. Some authors have focused on both early and late events in the peripheral immune system during stroke in mice and have observed an early activation of splenocytes that conceivably could result in immune-mediated damage in the developing CNS lesion,^37^ followed by global immunosuppression that affects the spleen, thymus, lymph nodes, and circulation that has been reported as mediated by a stroke-induced apoptosis of CD4+CD28+ cells in lymphoid organs^37,38^

Nevertheless, it is conceivable that CD4+ D28 null subset of T-cells could be resistant to stroke-induced apoptosis. CD4+ T-cells deficient in CD28 expression and compared with their CD28+ counterparts, they produce significantly higher levels of IFN-γ giving them the ability to function as proinflammatory cells. Moreover CD4+ CD28null T cell are highly olygoclonal and clones persist for years in circulation.^36^ Longevity of these cells appears to be related to their relative resistance to spontaneous cell death even in the absence of IL-2.^39^ This phenomenon is associated with low levels of expression of the a-chain of the IL-2R (IL-2Ra), despite their ability to produce large amounts of IL-2, and an increased expression of the anti-apoptotic molecule Bcl-2.^40–43^

In conclusion, we provided evidence of a higher peripheral percentage of some subsets of T-lymphocyte cells in subjects with acute ischemic stroke, a significant association with neurological deficit degree, and a predictive role of CD28 null cell peripheral percentage toward stroke diagnosis and TOAST subtype.
