# Peripheral Frequency of CD4+ CD28− Cells in Acute Ischemic Stroke: Relationship With Stroke Subtype and Severity Markers: Erratum

**DOI:** 10.1097/01.md.0000466908.35208.7e

**Published:** 2016-06-19

In the article “Peripheral Frequency of CD4+ CD28− Cells in Acute Ischemic Stroke: Relationship With Stroke Subtype and Severity Markers”,^1^ which appeared in Volume 94, Issue 20 of *Medicine*, Dr. Rosario Squatrito's name was spelled incorrectly. Also, reference 14 was partially incorrect. The article has since been corrected online.
